# A Reference DNA Barcode Library for UK Fungi associated with Bark and Ambrosia Beetles

**DOI:** 10.1038/s41597-025-05845-5

**Published:** 2025-09-29

**Authors:** Angelina Ceballos-Escalera, Theo Llewellyn, John Richards, Daegan Inward, Alfried Vogler

**Affiliations:** 1 Leverhulme Centre for the Holobiont, Department of Life Sciences, Imperial College London, Silwood Park Campus, Ascot, Berkshire, SL5 7PY UK; 2 Department of Life Sciences, Natural History Museum, Cromwell Road, London, SW6 7BD UK; 3 Forest Research, Alice Holt Research Station, Farnham, Surrey GU10 4LH UK

**Keywords:** Biodiversity, Fungal ecology

## Abstract

Bark and ambrosia beetles are ecologically important and widespread forest organisms. While flying between host plants and tunnelling into bark, they vector a high diversity of plant and insect-associated fungi within and between forests. These fungal communities live under the bark and rarely produce visible spore-bearing structures, making them difficult to sample and identify at scale. By combining passive insect trapping across the United Kingdom with individual beetle metabarcoding, we generated a reference DNA barcode library for fungi associated with over 1000 beetles, representing 25 native weevil species in the subfamily Scolytinae (Curculionidae). Sampling sites span longitudinal and latitudinal gradients, multiple forest types, and both natural and plantation forests. We use state-of-the-art fungal ITS2 OTU clustering and identification, resulting in 5274 identified fungal OTUs. This fungal diversity data can be used alongside extensive site and sample metadata to explore geographic and ecological biodiversity patterns. As these associations can be highly invasive and damaging, this resource provides a baseline to understand current fungal communities and monitor future changes.

## Background & Summary

Bark and ambrosia beetle-fungal symbioses are central to healthy forests, contributing essential ecosystem services, including wood decomposition and nutrient cycling^1^. However, some symbioses pose a serious threat, with mass outbreaks upsetting ecosystem balance^2^. Additionally, bark and ambrosia beetles often vector phytopathogenic fungi, contributing to the spread of devastating forest dieback^3–6^.

Beetle-associated fungi form diverse and complex communities. Fungal isolation and culturing have allowed us to describe in detail the main fungal symbionts of these systems, However, these fungi represent only a fraction of the associated fungal diversity. Whilst flying between host plants and tunnelling into bark, these beetles both actively and inadvertently pick up a wide range of fungi including endophytes, wood saprotrophs and lichens^7–9^. Hence, these beetles could be considered ‘samplers’ of plant and insect-associated fungal diversity for a given habitat. Passive insect collection traps, such as flight interception or Malaise traps, followed by whole beetle metabarcoding can therefore provide a high-throughput method for assessing the diversity of fungal communities that are otherwise difficult to study in a comprehensive and standardised manner.

Internal Transcribed Spacer (ITS) metabarcoding partners well with this sampling approach as it allows us to describe whole fungal community diversity, including unculturable or less dominant taxa^10–13^. Analysing fungal metabarcode data alongside sample metadata has identified the main drivers of fungal community composition at global scales, which include climate, biogeography and anthropogenic activity^14–17^. Furthermore, metabarcoding has revealed that many clades of fungi do not follow the traditional latitudinal diversity gradients observed in plants and animals^18,19^.

Many published bark and ambrosia beetle-fungal datasets focus on a single beetle species or genus, often the most aggressive invasive pests, neglecting the fungal communities associated with native, non-harmful beetles. As vector species strongly affects fungal community diversity and composition^9,20–24^, biodiversity surveys should aim to cover a wide range of vectors, especially rare and endemic beetle taxa.

Forest structure and plant composition significantly affect beetle-fungal diversity too^25–27^. This also applies to human-managed systems, with forest management strategy directly influencing bark beetle populations^28,29^. The effect of forest management on fungal communities is less well understood, however^30,31^. Therefore, datasets should also cover a range of host plant species and forest compositions or management strategies.

**Fig. 1:**
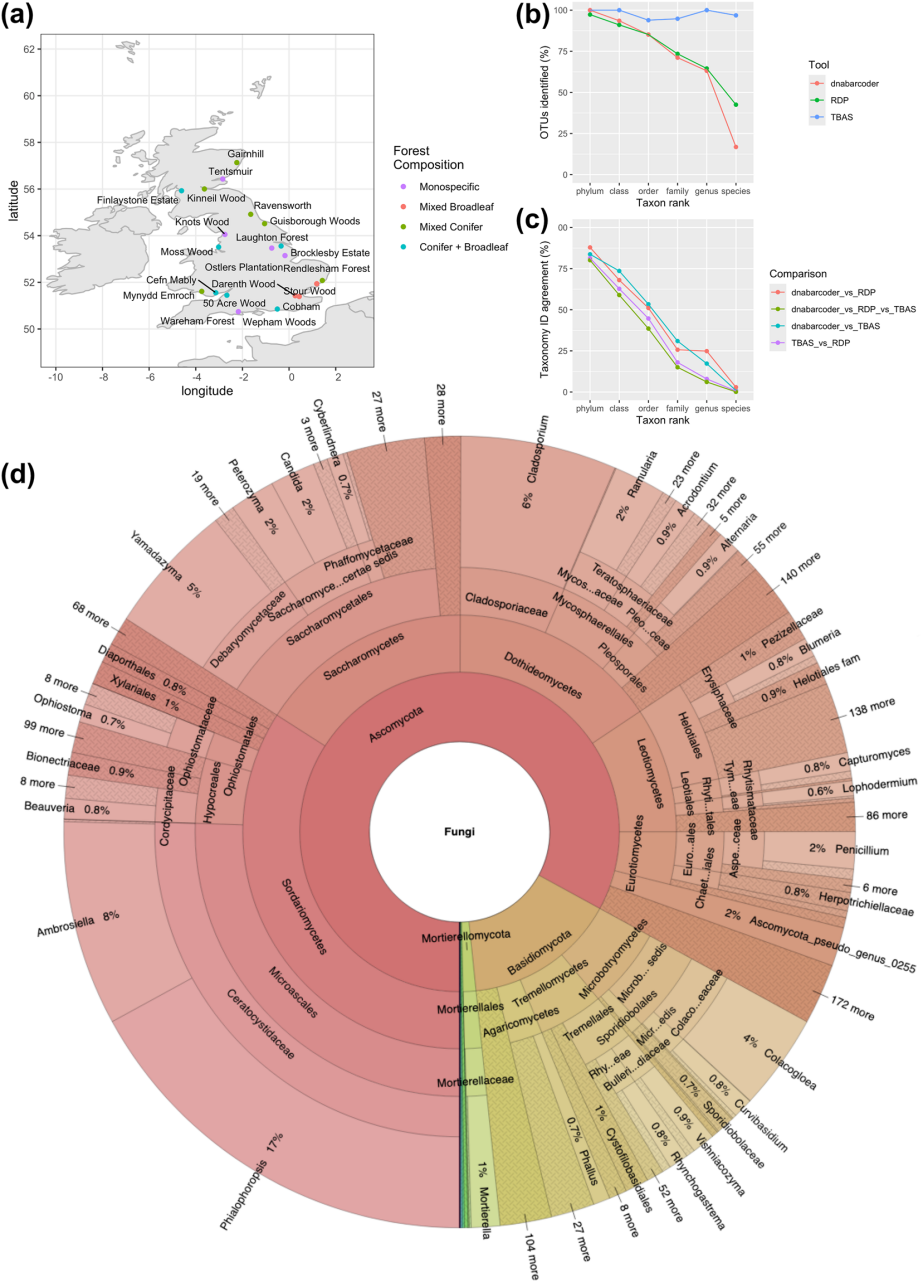
Summary of the distribution and taxonomy of fungal ITS2 OTUs associated with bark and ambrosia beetles collected in a UK biodiversity survey. (**a**) UK map showing locality and forest composition type of all 20 sites. (**b**) The proportion of OTUs identified at each taxonomic rank for three alternative identification tools. (**c**) The level of agreement in taxon IDs between the three identification tools at each taxon rank. (**d**) Krona plot showing the taxonomy of all fungal OTUs. An interactive version of this can be found on Figshare 10.6084/m9.figshare.29505365.

Here we present a national biodiversity survey of fungal communities associated with bark and ambrosia beetles across the United Kingdom. Our sampling sites cover the length and breadth of the country, providing longitudinal and latitudinal climatic gradients (Fig. 1a). We sampled at 20 sites with four forest composition types: monospecific conifers, mixed conifers, mixed broadleaves, and mixed conifer and broadleaves. For each site, we recorded primary and secondary tree species, altitude, planting date for plantations, and extensive climatic data (see Figshare Supplementary Data). In total, we collected 1064 bark and ambrosia beetle specimens, which we identified to 25 species in the weevil subfamily Scolytinae (Curculionidae) (Table 1).

**Table 1:** Overview of the beetle specimens collected in this study.

Beetle species	No. specimens	Forest Composition			
		Monospecific Conifers	Mixed Conifers	Mixed Broadleaves	Conifer + Broadleaves
*Anisandrus dispar*	23	X		X	X
*Cryphalus asperatus*	28	X	X		X
*Dryocoetes autographus*	89	X	X	X	X
*Dryocoetes villosus*	4	X		X	
Gnathotrichus materiarius*	1	X			
*Hylastes ater*	65	X	X		X
*Hylastes attenuatus*	61		X	X	X
*Hylastes brunneus*	75	X	X		X
*Hylastes cunicularius*	19		X		X
*Hylastes opacus*	51	X	X	X	X
*Hylesinus varius*	33	X	X		X
*Hylurgops palliatus*	178	X	X	X	X
*Orthotomicus laricis*	13	X	X		X
*Pityogenes bidentatus*	5	X	X		X
*Pityopthorus pubescens*	5	X	X		X
*Polygraphus poligraphus*	14		X		X
*Tomincus minor*	12	X	X		X
*Tomicus piniperda*	159	X	X		X
*Trypodendron domesticum*	29	X	X	X	X
*Trypodendron lineatum*	83	X	X		X
*Trypodendron signatum*	2	X			X
*Xyleborinus saxesenii*	91	X	X	X	X
*Xyleborus monographus*	1				X
Xylosandrus germanus*	20			X	

Crosses mark which forest composition types each species was found in. Taxa marked with * are naturalised invasive beetles first detected in a UK survey between 2013 and 2017^36^.

Merging forward and reverse reads and quality filtering resulted in 12,210,693 total paired reads (mean 12,158 paired reads per sample). Dereplication reduced this to 1,591,669 unique sequences, and singleton removal left 119,015. Removing low abundance sequences (<4 reads) and clustering produced 11,991 zero percent ASVs (zOTUs). Chimera detection removed 385 ASVs, resulting in a final dataset of 11,606 unique Amplicon Sequence Variants (ASVs), of which 7,896 were identified as Fungi. We performed Basic Local Alignment Search (BLAST) for all fungal ASVs against the UNITE ITS2 database and then assigned them to the taxon of the top BLAST hit if the percentage sequence similarity exceeded a taxon-specific sequence-similarity threshold calculated by dnabarcoder (see reference Vu *et al*.^32^ for method details). Using these taxon-specific sequence similarity thresholds, we identified 6,939 ASVs to at least phylum level, 6,463 to at least class, 5,893 to order, 4,978 to family, 4,459 to genus and 1,214 to species.

We dynamically clustered ASVs into Operational Taxonomic Units (OTUs) using the aforementioned taxon-specific cut-offs (see Methods) resulting in 5,303 OTUs. We removed any OTUs with more than one read in negative controls, leaving a final dataset of 5,274 fungal OTUs. Of these, 100% were identified to phylum level, 94% to class, 85% to order, 71% to family, 63% to genus, and 17% to species. We compared our identifications, which follow current best practices in fungal sequence identification, to two commonly used identification tools (the Ribosomal Database Project (RDP) Classifier and the Tree-Based Alignment Selector Toolkit (T-BAS)^33^). This showed that current best practices are generally more conservative at species level, assigning taxon names to a lower proportion of OTUs (Fig. 1b). Above species level dnabarcoder identifies a similar proportion of OTUs to RDP but fewer OTUs than T-BAS. Despite T-BAS identifying a higher proportion of taxa, the actual assignments differ drastically to RDP, demonstrating a lack of consistency in the results of commonly used tools (Fig. 1c).

The average number of OTUs per beetle was 65 ± 38, with a range from 0 to 209. The median number of OTUs per beetle was 59, with an interquartile range of 38–87. The average number of beetles a fungal OTU occurs in (prevalence) was 13 ± 36 with a range from 1–730.

The taxonomic identifications of all fungal OTUs are shown in Fig. 1d. The Ascomycota is the most abundant phylum making up 83% of all OTUs reads. The five most abundant classes were Sordariomycetes (34%), Saccharomycetes (16%), Dothideomycetes (16%), Leotiomycetes (9%), and Eurotiomycetes (4%) and in the Basidiomycota Microbotryomycetes (6%) and Tremellomycetes (5%). The most abundant genera were *Phialophoropsis* and *Ambrosiella*, representing 17% and 8% of all OTU reads, respectively. The five most abundant OTUs identified to species level were *Ambrosiella hartigii* (5% of all OTU reads), *Colacogloea falcata* (4%), *Ambrosiella grosmanniae* (3%), *Yamadazyma tenuis* (2%), and *Peterozyma toletana* (2%) (see Figshare Supplementary Data for interactive taxonomic Krona chart with abundance data). *Ambrosiella*, *Yamadazyma* and *Peterozyma* are all well-known bark beetle associates^1,34^.

**Fig. 2:**
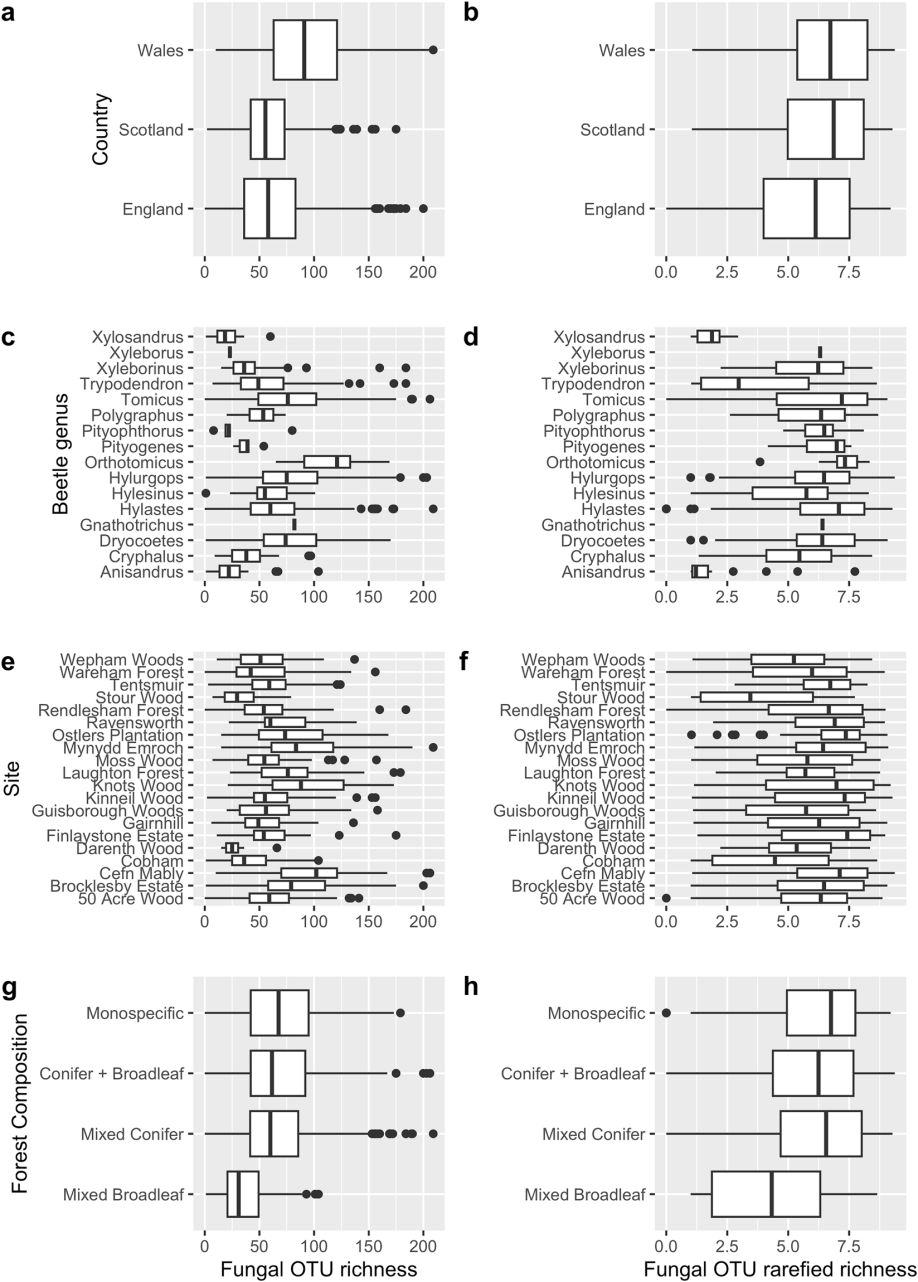
Boxplots summarising fungal OTU richness across four selected metadata variables. Plots in left column show raw OTU richness (i.e. total number of OTUs per sample). Plots in right column show rarefied richness to 10 sequencing reads, calculated using vegan’s rarefy function. Metadata variables are country (**a,b**), host beetle genus (**c,d**), sample site (**e,f**), and forest composition (**g,h**).

To facilitate future ecological studies, we provide fungal OTU richness per sample (raw and rarefied) alongside extensive sample metadata to allow future comparisons of fungal diversity between host beetle taxa, and site-based variables. Figure 2 shows an example of this for four selected metadata variables. We also provide an OTU x sample table to allow for beta-diversity analyses.

This dataset provides a solid baseline for biodiversity monitoring, which can be compared to future data to track temporal trends in fungal diversity and forest health as sampled through beetles. This has implications for fungal conservation as it will allow us to identify sites of high or unique diversity. Additionally, analysing this data alongside its detailed metadata will allow us to evaluate the influence of abiotic and biotic drivers on fungal and beetle community diversity. Finally, a comprehensive survey of the fungi associated with native beetle species will allow us to identify whether invasive beetles are also bringing new fungi into the UK. As bark beetles commonly vector phytopathogenic fungi, this will help us to track and control future forest disease outbreaks. Equally, though benign in the UK, these beetle-fungal symbioses may become invasive pests outside their native range. Therefore, understanding what fungi these beetles normally carry will help other countries to develop more effective biosecurity monitoring plans.

## Methods

### Sample collection

We selected 20 sampling sites across the UK, which we classified into four forest composition types: monospecific, mixed conifers, mixed broadleaves, and conifer + broadleaves (Fig. 1a). Monospecific forests consisted of only Scots pine (*Pinus sylvestris* L.) or Corsican pine (*Pinus nigra* Arnold). Mixed conifer sites contained one of the *Pinus* species alongside Sitka spruce (*Picea sitchensis* (Bong.) Carr.). Mixed broadleaf forests consisted of multiple broadleaf species, including but not limited to sweet chestnut (*Castanea sativa* Mill.), pedunculate oak (*Quercus robur* L.), beech (*Fagus sylvatica* L.) hornbeam (*Carpinus betulus* L.) and sessile oak (*Quercus petraea* (Matt.) Liebl.). Sites dominated by conifers but with a smaller proportion of broadleaves were classified as conifer + broadleaves. Within each site, we placed Lindgren multiple-funnel traps (^35^, Phero Tech), spaced 50 metres apart along a 250 metre transect. Traps were baited with 100% ethanol + a-pinene to mimic volatile chemicals emitted by dying trees^36^. The base of the traps was filled with propylene glycol (65% concentration) to preserve beetles and traps were emptied every two weeks from April to August 2017.

After being removed from the traps, samples were immediately transferred to pure ethanol and frozen at −80 °C to preserve DNA quality. We morphologically identified all beetle specimens. When more than eight specimens from a single site belonged to the same species, we randomly selected minimum eight up to a maximum of 12 specimens to represent that species at that site. For rare species (<eight specimens/species/site), one specimen per species per site was selected.

### Fungal metabarcoding DNA extraction, PCR and sequencing

We rinsed samples with pure water and placed each sample in individual 1.5 ml Eppendorf tubes. We broke down samples completely using sterile pestles and then DNA was extracted using the QIAGEN DNA easy blood and tissue spin column extraction kit (Qiagen, Valencia, CA, USA) according to the manufacturer’s instructions and eluted in 200 µl of AE buffer.

We chose to amplify the fungal ITS2 region due its limited internal length variation and used primers ITS86F (5′- GTGAATCATCGAATCTTTGAA-3′) and ITS4 (5′-TCCTCCGCTTATTGATATGC-3′)^37,38^. We added unique six-letter primer tags to allow pooling and demultiplexing of samples. To avoid tag-jumping we used the same tag for forward and reverse primers for each specimen (twinned tags) and only kept amplicons with F1-R1, F2-R2 etc. The PCR reaction mix contained 2.5 µl of TaKaRa buffer™, 0.5 µl dNTPs (Bioline), 1 µl of each primer, 0.1 µl of TaKaRa Taq™, 18 µl ddH_2_O, and 2 µl of DNA template. PCR cycle conditions were: 3 minutes at 94 °C, 34 denaturing-annealing cycles of 45 seconds at 94 °C, 55 seconds at 55 °C, 1.5 minutes at 72 °C, and finally 10 minutes at 72 °C. For each sample, three independent PCR reactions were performed from the original DNA extract at three annealing temperatures (54 °C, 55 °C and 56 °C) to account for PCR stochasticity^12^. Each plate contained a blank negative control to ensure no contamination occurred during extraction, PCR or sequencing. PCR products were verified using gel electrophoresis on a 2% agarose gel. Positive control mock community tests were not performed.

PCR products were purified to eliminate fragments < 200 bp using the Agencourt AMPure XP purification kit, following protocol 000387v001. We pooled the tagged, amplified DNA in equal proportions into a 96-well plate. Library preparation, homogenisation and indexing were performed at the sequencing facility using Nextera XT with a second PCR. Libraries were sequenced on an Illumina HiSeq 2500 platform with version 2 chemistry (Rapid mode), using sequencing by synthesis (SBS) technology to generate 2 × 250 bp paired-end reads.

### Sequence denoising

We demultiplexed the 96 libraries using Illumina software to remove indices, and then used Cutadapt^39^ to remove primer sequences. The following steps were all performed via VSEARCH v2.30.0; first we merged forward and reverse reads and removed singletons using the fastx_uniques command. Following this, we removed sequences with less than four reads using the unoise3 algorithm and then removed chimeras using the UCHIME3 algorithm via the uchime3_denovo command^40^. Finally, we mapped all quality-filtered, chimaera-removed, merged reads against each other at 100% nucleotide identity to produce a list of ASVs in each sample using usearch_global (i.e. zero-radius Operational Taxonomic Units or zOTUs).

### Fungal sequence taxonomic identification

To get an overview of which fungi were present in our dataset, we assigned taxonomy to fungal ASVs using the dnabarcoder pipeline^32^. First, we used ITSx to trim sequences to just the target ITS2 region^41^. Using ITSx’s curated group-specific Hidden Markov Models, we then retained sequences whose closest match was the Fungi HMM. We then BLASTed each ASV against an ITS2 extracted version of the UNITE + INSD 2024 reference database^42^ using BLASTn^43^. The ITS2 reference database was downloaded from here: https://zenodo.org/records/13336328. Based on the best BLAST hits we then assigned sequences to taxonomic groups. Rather than relying on a fixed similarity threshold to assign taxa, we used the dnabarcoder taxon-specific sequence similarity thresholds calculated for each taxon in the UNITE reference database (ITS2 cut-offs can be downloaded here: https://github.com/vuthuyduong/dnabarcoder/blob/master/data/UNITE_2024_cutoffs/unite2024ITS2.unique.cutoffs.best.json).

### OTU clustering and diversity

As fungal ITS sequences can vary both between individuals of a species and between gene copies of a single genome, we clustered ASVs into OTUs to better approximate species diversity^44^. To avoid arbitrary OTU sequence clustering thresholds, we implemented the dynamic clustering approach of Florence *et al*.^10^. To summarise, this method uses the dnabarcorder identified ASVs as cluster cores for closed-reference clustering, matching unidentified ASVs to these cores using the dnabarcoder taxon-specific cut-offs and BLASTn^10^. This is followed by de novo clustering of any unclustered ASVs via BLASTClust^45^, which are given unique IDs based on the lowest identified taxonomic rank. Florence *et al*.^10^ describe this as a nested clustering approach, from kingdom to species level, constraining each stage by the results of its supertaxon (e.g. Ophiostomatales clustering constrained by Sordariomycetes results, constrained by Ascomycota results). We removed any OTUs with more than one read in any of the negative controls.

We compared the OTU taxonomy obtained from dnabarcoder to two alternative methods: 2) the Bayesian classifier RDP trained on UNITE, and 2) phylogenetic placement using the Tree-Based Alignment Selector (T-BAS) toolkit v2.3 with the Fungi v3 reference phylogeny^33^. Both methods used default settings, and for T-BAS, we used RAxML’s evolutionary placement algorithm (EPA) as the placement method^46^.

We calculated presence-absence based fungal OTU richness per sample. We also provide rarefied richness estimates to account for uneven sequencing depth. For rarefied richness, we used vegan v2.6-4’s rarefy function, subsampling to 10 reads based on an observed natural cut-off when plotting read abundance across all samples.

## Data Records

The processed sequence data and metadata are available on the public NCBI SRA under SRP accession SRP600814, BioProject accession PRJNA1291454^47^. All other data files are available at Figshare 10.6084/m9.figshare.29505365^48^. Figshare contains: (1) The OTU x Sample table, (2) Full Taxonomic Identifications for each OTU as determined by dnabarcoder and BLAST against UNITE and subsequent dynamic clustering, (3) An interactive KRONA plot showing taxonomy and abundance of OTUs, (4) The ASV x Sample table, (5) Representative ITS2 sequences for all ASVs in FASTA format, (6) Representative ITS2 sequences for all OTUs in FASTA format, (7) Sample metadata, (8) site metadata, (9) The OTU identifications of T-BAS and RDP, (10) the OTU alpha diversity statistics calculated for each beetle sample, and (11) the taxon-specific sequence similarity thresholds used for dnabarcoder ASV identification and dynamic OTU clustering.

## Technical Validation

All extractions, PCRs and sequencing runs were performed with negative controls to ensure no lab contamination occurred. None of the negative controls showed electrophoresis bands after extraction or PCR. In total, 29 OTUs had more than one read in any negative control and were thus removed from the OTU dataset. This highlights the importance of sequencing negative controls even if they show no electrophoresis bands. Our use of paired unique primer tags for all samples (twinned tags) allowed us to avoid tag jumping issues as only amplicons with matching forward and reverse primers were retained for analysis.

As there is ongoing debate about whether to use ASVs or OTUs in fungal metabarcoding^13,32,44,49^, we have made both datasets available for our sequences. However, given the known phenomena of intragenomic and intraspecific ITS variation, we believe the dynamically clustered OTUs most closely represent fungal species diversity for future studies wishing to analyse diversity patterns.

Bark and ambrosia beetles may encounter transient environmental fungi in the bark and whilst flying between plants. As such, there is a risk that fungal metabarcodes of these beetles may be dominated by generalist environmental fungi and could miss important mutualistic symbionts. When assessing the taxonomy of our OTUs, this does not appear to the case in this dataset as our most abundant OTUs are known beetle associates (Fig. 1d). To supplement this, we also performed a non-exhaustive literature review to broadly assess whether fungal OTUs within the two main orders of bark beetle fungal symbionts (Microascales and Ophiostomatales) associate with similar beetles to previous studies. Of the 32 Microascales and Ophiostomatales OTUs identified to species level, 12 have been reported in other studies to be associated with beetles of the same species or genus as those observed in this dataset (see Table 2). This validates that the fungal communities in this dataset make sense given the range of host beetles and are not purely random environmental taxa.

**Table 2:** Microascales & Ophiostomatales fungal species identified within the OTUs, with their reported bark beetle hosts.

Fungal species	Beetle hosts (this study)	Beetle hosts (literature)	Prevalence	Refs
*Ambrosiella grosmanniae*	*Anisandrus dispar, Dryocoetes autographus, Hylastes ater, Hylastes attenuatus, Hylastes brunneus, Hylastes opacus, Hylesinus varius, Hylurgops palliatus, Tomicus piniperda, Trypodendron lineatum, Xyleborinus saxesenii, Xylosandrus germanus*	*Xylosandrus germanus*	68	^50^
*Ambrosiella hartigii*	*Anisandrus dispar, Cryphalus asperatus, Dryocoetes autographus, Hylastes ater, Hylastes attenuatus, Hylastes brunneus, Hylastes opacus, Hylesinus varius, Hylurgops palliatus, Pityogenes bidentatus, Polygraphus poligraphus, Tomicus minor, Tomicus piniperda, Trypodendron domesticum, Trypodendron lineatum, Xyleborinus saxesenii, Xylosandrus germanus*	*Anisandrus dispar, X. germanus, Tomicus piniperda*	123	^51^
*Cephalotrichum brevistipitatum*	*Hylurgops palliatus*		1	
*Cephalotrichum nanum*	*Hylastes brunneus, Hylurgops palliatus, Tomicus piniperda, Trypodendron domesticum*		7	
*Ceratocystis grandicarpa*	*Xyleborinus saxesenii*		5	^50,52^
*Ceratocystis minor*	*Dryocoetes autographus, Hylastes brunneus*	*Dendroctonus frontalis*	2	^53^
*Ceratocystis paradoxa*	*Dryocoetes autographus, Tomicus piniperda*		3	
*Ceratocystis piceae*	*Cryphalus asperatus, Dryocoetes autographus, Dryocoetes villosus, Gnathotrichus materiarius, Hylastes attenuatus, Hylastes brunneus, Hylastes opacus, Hylesinus varius, Hylurgops palliatus, Polygraphus poligraphus, Tomicus piniperda, Trypodendron domesticum, Trypodendron lineatum, Xyleborinus saxesenii, Xyleborus monographus*		71	
*Chalaropsis ovoidea*	*Dryocoetes autographus, Hylurgops palliatus, Trypodendron lineatum, Xyleborinus saxesenii*		7	
*Endoconidiophora pinicola*	*Hylastes brunneus*		2	
*Graphilbum fragrans*	*Dryocoetes autographus, Hylastes ater, Hylastes attenuatus, Hylastes brunneus, Hylastes cunicularius, Hylastes opacus, Hylurgops palliatus, Orthotomicus laricis, Tomicus piniperda, Trypodendron domesticum, Trypodendron lineatum, Trypodendron signatum, Xyleborinus saxesenii, Xylosandrus germanus*	*Tomicus minor, Trypodendron domesticum*	121	^54,55^
*Grosmannia huntii*	*Dryocoetes autographus, Hylurgops palliatus, Trypodendron lineatum*	*Dendroctonus ponderosae, Ips pini, Hylastes macer, Tomicus piniperda, Hylastes ater, Dendroctonus terebrans, Hyalstes tenuis, Hylastes salebrosus, Hylobius pales*	11	^56–60^
*Leptographium cucullatum*	*Cryphalus asperatus, Dryocoetes autographus, Hylastes opacus, Hylurgops palliatus, Orthotomicus laricis, Tomicus piniperda, Trypodendron lineatum*	*Dryocoetes autographus*	16	^55^
*Leptographium piceaperdum*	*Cryphalus asperatus, Dryocoetes autographus, Hylesinus varius, Hylurgops palliatus, Polygraphus poligraphus, Tomicus piniperda, Trypodendron domesticum, Trypodendron lineatum*	*Dryocoetes hectographus, Dryocoetes autographus, Cryphalus montatus, Cryphalus piceae, Polygraphus proximus*	51	^61^
*Leptographium taigense*	*Polygraphus poligraphus*	*Dryocoetes autographus, Hylurgops palliatus*	3	^55^
*Leptographium tardum*	*Trypodendron domesticum*	*Trypodendron domesticum, Trypodendron signatum, Dryocoetes alni*	2	^62^
*Leptographium vulnerum*	*Dryocoetes autographus, Hylastes cunicularius, Tomicus piniperda, Trypodendron lineatum*		6	
*Ophiostoma australiae*	*Trypodendron domesticum*		1	
*Ophiostoma canum*	*Cryphalus asperatus, Dryocoetes autographus, Gnathotrichus materiarius, Hylastes ater, Hylastes attenuatus, Hylastes brunneus, Hylastes opacus, Hylurgops palliatus, Orthotomicus laricis, Tomicus minor, Tomicus piniperda, Trypodendron domesticum, Trypodendron lineatum, Xyleborinus saxesenii*	*Tomicus minor, Hylastes brunneus and Hylurgops palliatus, Tomicus piniperda, Trypodendron lineatum*	171	^55,63^
*Ophiostoma denticulatum*	*Dryocoetes autographus, Hylastes ater, Hylastes attenuatus, Hylastes brunneus, Hylastes opacus, Hylurgops palliatus, Orthotomicus laricis, Pityogenes bidentatus, Tomicus minor, Tomicus piniperda, Trypodendron lineatum, Xyleborinus saxesenii*	*Scolytus ratzeburgi*	45	^64^
*Ophiostoma distortum*	*Tomicus piniperda, Trypodendron domesticum*	*Trypodendron domesticum*	4	^54^
*Ophiostoma karelicum*	*Hylesinus varius, Trypodendron domesticum*	*Tomicus minor*	8	^55^
*Ophiostoma novo-ulmi*	*Trypodendron domesticum*	*Scolytus multistriatus, Hylurgopinus rufipes, Scolytus scolytus*	2	^65^
*Ophiostoma penicillatum*	*Hylastes attenuatus, Hylastes brunneus, Hylastes cunicularius, Hylesinus varius, Hylurgops palliatus, Tomicus piniperda, Trypodendron lineatum*	*Hylastes ater*	16	^55^
*Ophiostoma tapionis*	*Cryphalus asperatus, Dryocoetes autographus, Hylurgops palliatus*	*Hylastes brunneus, Hylurgops palliatus, Pityogenes*	5	^55^
*Ophiostoma villosum*	*Dryocoetes villosus, Trypodendron domesticum*	*Dryocoetes villosus*	3	^66^
*Sporothrix aurorae*	*Hylastes opacus, Hylurgops palliatus, Tomicus piniperda*		4	
*Sporothrix eucastaneae*	*Anisandrus dispar, Hylesinus varius, Polygraphus poligraphus, Xyleborinus saxesenii, Xylosandrus germanus*	*Scolytus intricatus, Anisandrus dispar, Xyleborus monogrpahus*	14	^54^
*Sporothrix fusiformis*	*Anisandrus dispar, Hylastes ater, Hylesinus varius, Hylurgops palliatus, Orthotomicus laricis, Tomicus minor, Tomicus piniperda, Trypodendron domesticum, Xyleborinus saxesenii*		23	
*Sporothrix inflata*	*Dryocoetes autographus, Hylastes ater, Hylastes attenuatus, Hylurgops palliatus*	*Scolytus intricatus*	7	^54^
*Sporothrix stenoceras*	*Xyleborinus saxesenii*		1	
*Spumatoria longicollis*	*Dryocoetes autographus, Hylastes ater, Hylastes attenuatus, Hylurgops palliatus, Orthotomicus laricis, Tomicus piniperda, Trypodendron lineatum, Xyleborinus saxesenii*		15	

Prevalence = number samples detected in here.

## Data Availability

The data generated and presented in this study are available in the following repositories; processed sequence data and metadata are available on the public NCBI SRA under SRP accession SRP600814, BioProject accession PRJNA1291454^47^, All other data files are available at Figshare 10.6084/m9.figshare.29505365^48^.

## Code availability

All code used to process sequences, identify ASVs, cluster them into OTUs and plot the results can be found at: https://github.com/theo-llewellyn/UK_Survey.
